# Implementing a Smart Method to Eliminate Artifacts of Vital Signals

**Published:** 2015-12-01

**Authors:** A. Javadpour, A. Mohammadi

**Affiliations:** 1 Neuroscience Research Center, Baqiyatallah University of Medical Sciences, Tehran, Iran

**Keywords:** Brain Signals, Artifact, Noise Elimination, Digital Circuit’s Complex

## Abstract

**Background:**

Electroencephalography (EEG) has vital and significant applications in different medical fields and is used for the primary evaluation of neurological disorders. Hence, having easy access to suitable and useful signal is very important. Artifacts are undesirable confusions which are generally originated from inevitable human activities such as heartbeat, blinking of eyes and facial muscle activities while receiving EEG signal. It can bring about deformation in these waves though.

**Objective:**

The objective of this study was to find a suitable solution to eliminate the artifacts of Vital Signals.

**Methods:**

In this study, wavelet transform technique was used. This method is compared with threshold level. The threshold intensity is efficiently crucial because it should not remove the original signal instead of artifacts, and does not hold artifact signal instead of original ones. In this project, we seek to find and implement the algorithm with the ability to automatically remove the artifacts in EEG signals. For this purpose, the use of adaptive filtering methods such as wavelet analysis is appropriate. Finally, we observed that Functional Link Neural Network (FLN) performance is better than ANFIS and RBFN to remove such artifacts.

**Results:**

We offer an intelligent method for removing artifacts from vital signals in neurological disorders.

**Conclusion:**

The proposed method can obtain more accurate results by removing artifacts of vital signals and can be useful in the early diagnosis of neurological and cardiovascular disorders.

## Introduction

**Figure 1:**
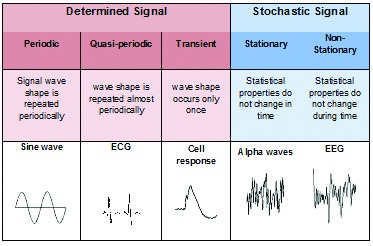
Categorization of Signals

Signals produced by body organs are combined with each other or affected by noise. Vital signal processing refers to separating objective signals from mixed and noised signals and then extraction of suitable parameters of a signal [1]. Deterministic signals are signals whose wave forms of signals are determined and clear, and they are completely predictable [2]. Deterministic signals are in three categories: periodic, quasi-periodic and transient. Random or statistical signals are created due to random and uncoordinated depolarization of a group of cells such as neural cells of brain producing EEG signals (Electroencephalogram). Wave forms of such signals are undetermined and can only be explained using statistical concepts. Based on the type of biological process, random signals are divided into two stationary and non-stationary categories [3]. In stationary random signals, statistical characteristics of signals do not change over time. If the biological process that produces random signal is in a special condition, the produced random signal would be non-stationary. For instance, the EEG of a patient who has undergone an epileptic sudden attack, is a non-stationary random signal [4]. Figure 1 shows these categories with their examples [5]. Recording electrical activities of brain (EEG) consists of so many internal and external disturbances, for instance ocular artifacts which are recorded simultaneously with brain signals due to ocular electrical activities [6]. Due to higher amplitude of ocular artifacts proportional to brain signals, many methods have been presented to reduce their effect [5, 7].

## Material and Methods

In this part, we will briefly review methods provided in this paper. One of the methods used in the present paper to eliminate EOG (Electrooculography) artifact from EEG signal is a method based on the conversion of wavelet. The other method is adaptive filter which can be very efficient due to its variability nature with the time of signals related to human body [8]. Although linear adaptive filters have many applications, these filters are not suitable for modelling non-linear problems. Since artifact signals go through a non-linear path, linear adaptive filters are not suitable for modelling them. Due to non-linear nature of this problem, an adaptive filter should be used which has high ability to model artifact signals [9].

**Figure 2:**
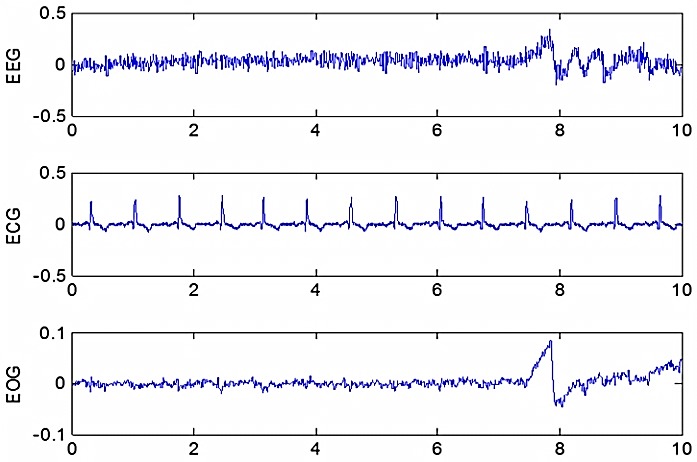
An example of used data provided from Phisionet website which consists of EEG signal along with EOG and ECG signals.

Neural networks and fuzzy interpretation systems are very suitable non-liner estimators which can be used. One of the methods, which can be used for this objective efficiently, is combining neural network and fuzzy systems called neuro-fuzzy network. That is why in this paper, we have used an adaptive fuzzy interpretation network, an adaptive network based on RBFN and an adaptive FLN-RBFN network [9-11]. Data used in this research were collected from Phisionet website [12]. The provided data were EEG signals recorded along with EOG and ECG artifacts. The data were used to test the mentioned programs in chapter three. Figure 2 indicates an example of the used data which consist of EEG signals along with EOG and ECG signals recorded separately. Duration of recording signal was 10 seconds.

### Neuro-fuzzy Adaptive Interpretation Network

**Figure 3:**
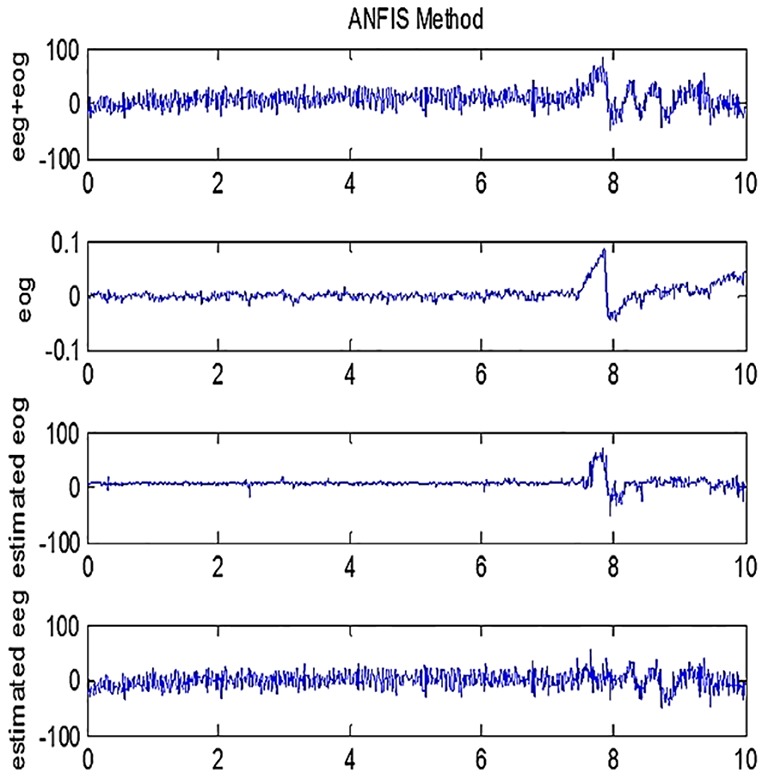
Result of Performing Neural-fuzzy Adaptive Interpretation Network

**Figure 4:**
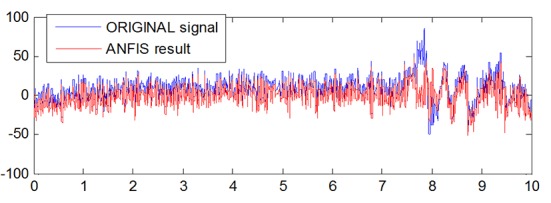
Result of Performing Neuro-fuzzy Adaptive Interpretation Network (red) along with Main Signal (blue)

This method of fuzzy interpretation was used to model artifact effects. EOG artifact signal was exerted as an input of this network. The output is an estimation that this network has gotten the effect of EOG artifact on EEG signal [13]. Our objective was to train this network to estimate the function added to EEG due to EOG. In this program, two inputs (EOG signal input and its delayed form) are applied. In Figure 3, the output of neuro-fuzzy adaptive interpretation network is indicated [10, 14]: EEG signals along with EOG artifact (Figure1), EOG recorded artifact (Figure 2), estimated artifact (Figure 3) and estimated EEG signals after elimination of EOG artifact (Figure 4). As indicated above, this network efficiently estimated and eliminated the effect of EOG. In Figure 4, the main signal is indicated along with signal after elimination of the artifact. Based on recorded EOG, the exact part which is related to EOG is estimated and its effect is eliminated.

**Figure 5:**
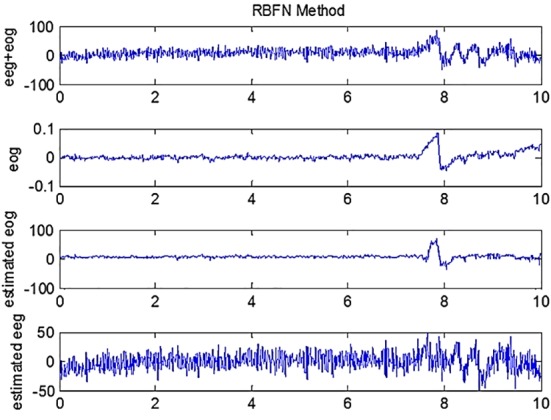
Result of Performing FLN-RBFN Network

**Figure 6:**
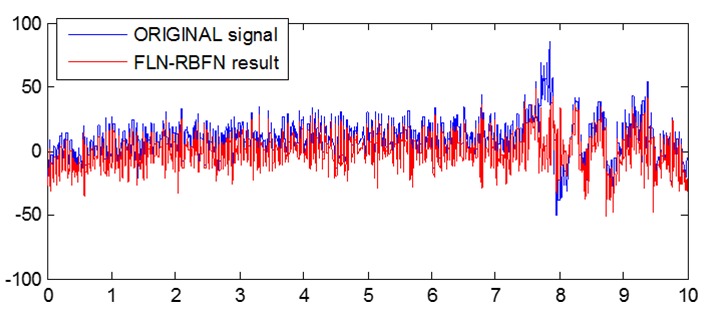
Result of Performing FLN-RBFN Network (red) along with Main Signal (blue)

In Figure 5, EEG signal is recorded along with EOG artifact, the effect of estimated artifact and estimated EEG signal can be observed after elimination of the effect of EOG. In Figure 6, the main signal can be observed simultaneously along with the signal after elimination of artifact. As seen below, based on recorded EOG, the exact part which is related to EOG is estimated and its effect is eliminated. This network has also diagnosed the effect of EOG artifact efficiently and has eliminated it.

**Figure 7:**
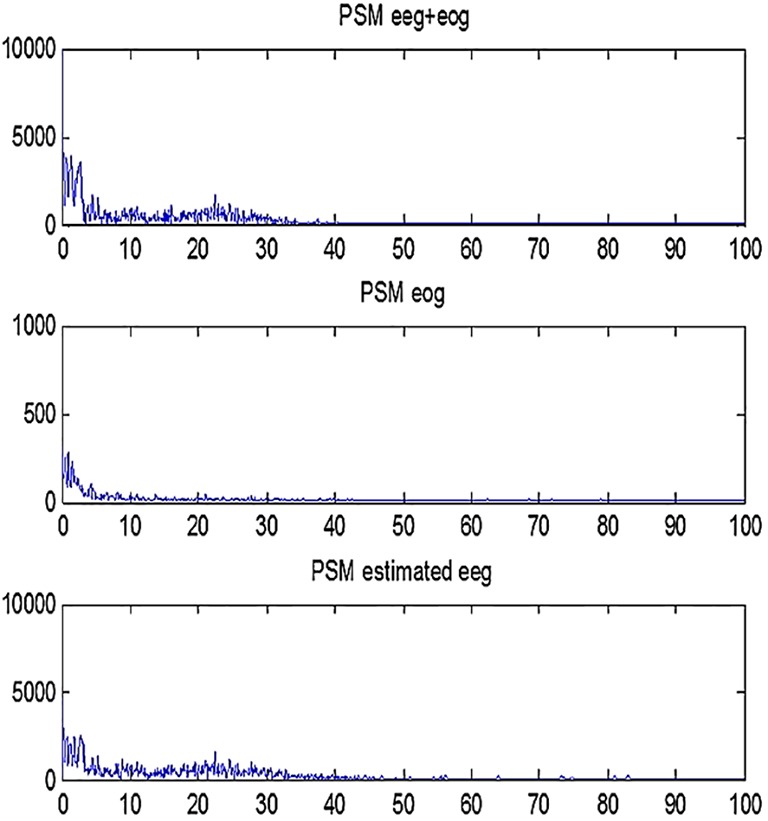
Result of Performing FLN-RBFN Network (red) along with Main Signal (blue)

In Figure 7, the amplitude of the signal power spectrum (PSM) is used for comparing. In Figure 5, we can observe PSM for signal along with artifact, PSM of artifact signal and PSM of signal after elimination of artifact [15, 16].

**Figure 8:**
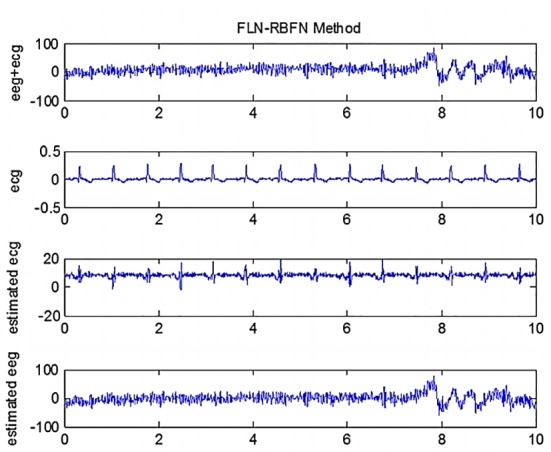
Result of Performing FLN-RBFN Network: EEG Signal along with ECG Artifact (Figure 1), Recorded ECG Artifact (Figure 2), Estimated Artifact (Figure 3) and Estimated EEG Signal after Elimination of ECG Artifact (Figure 4)

As you can see, the effect of parts with low frequency related to EOG artifact has been reduced after eliminating artifact in the signal. This method has also been applied to eliminate the effect of EOG artifact whose result is indicated in Figure 8. In the same way, ECG artifact has been applied as an input for this network. The output is also an estimation made based on the effect of ECG artifact on EEG signal. Then, this network estimated the effect of ECG artifact on EEG signal and its effect was eliminated from EEG signal. Moreover in this case, two inputs of ECG signals and its delayed forms are used.

In part 3 of Figure 8, the effect of existing ECG artifact can be observed in the estimated EEG. Correlation criteria are used to compare the effects of adaptive FLN-RBFN network performed to eliminate ECG artifact from EEG signal. In this regard, the correlation between signals along with artifact and ECG artifact were compared with the correlation of signals after elimination of artifact and ECG artifact as observed in Table 1.

**Table 1:** Considered Condition for Elimination

**Considered Condition**	**Correlation Value**
Before elimination of artifact	72.75
After elimination of artifact	1.4492 e-4

## Results

**Figure 9:**
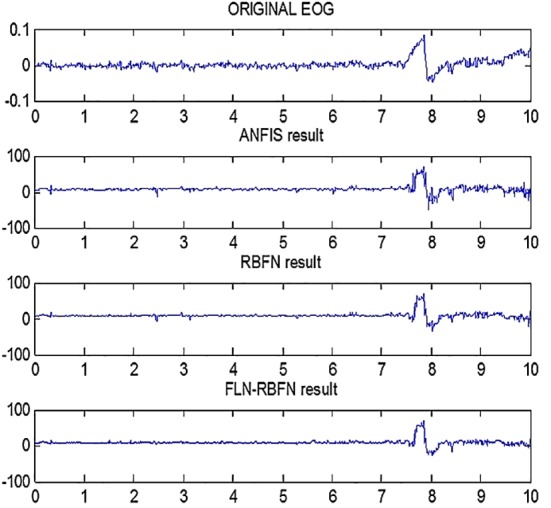
Effect of Estimated EOG Using Implemented Methods

**Figure 10:**
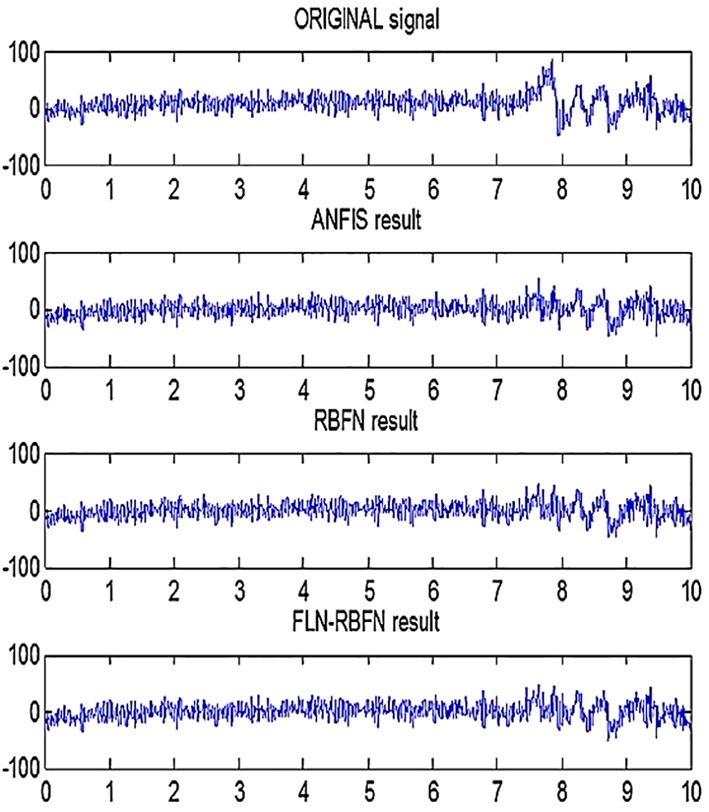
Output of all Methods

Figures 9 and 10 indicate the output of methods simultaneously. In Figure 9, the main EOG can be observed along with EOG estimation of these methods. Figure 10 also indicates EEG out of methods after elimination of noise. Compared with the form of the main signal, it can be observed that the artifact has been efficiently diagnosed and eliminated.

One of the ways to compare the results of implemented methods is to use MSE criteria. The following equation is used to compute these criteria:

y(k) and q(k) are real and efficient outputs of adaptive filter, respectively. The results of MSE for methods can be observed in Table 2. As seen in this table, the result of FLN-RBFN method is better than ANFIS and RBFN methods regarding MSE criteria, which also has better efficiency. As mentioned before, one advantage of this method was that there was no need to determine neurons from the beginning and the method itself determines the neurons. At the end, the designed FLN-RBF network is implemented on FPGA.

**Table 2:** Considered Condition for Elimination

**MSE**	**Number of Neurons**	**Filter Type**
137.8864	9	ANFIS
128.3132	9	RBFN
124.9235	8	FLN-RBFN

## Discussion and Conclusion

Brain signals (EEG) have critical and important applications in different medical fields. So, it is very important to access brain signals with high quality and adequate power. Meanwhile, the presence of interfering signals (artifacts) which affect the shape of EEG signal are inevitable. This interfering signal is always on the way of valuable EEG signal; it disrupts the ability to use it optimally. As mentioned under previous sections, artifacts are unwanted disturbances which are mainly derived from inevitable human activities such as heartbeat, blinking and facial muscle activity during the reception of EEG signals. They can deform waves and create ambiguity on it. Although adaptive linear filtering is widely used in electrical signal processing and filtering, the disturbances and interfering signals, these filters are not suitable for modelling non-linear problems. Given that artifact signals pass through a non-linear path, they are non-linear in nature and the linear adaptive filtering is not appropriate for modelling. Thus, a non-linear adaptive filtering should be used which has a high ability to model artifact signals. 

Shahabi et al. [13] have presented a method based on Fourier analysis of signal that uses the frequency spectrum to filter out EEG signal. In this study, high-pass linear filtering is discussed with an approach to remove some parts of EEG signal. In filtering process, the Fourier transform of signal is calculated to specify its spectrum. Then, the undesired frequency components of signals are removed, and finally by applying the inverse Fourier transform, the filtered signal is obtained in the time domain. Using this method results in the removal of original signal and noise which is not convenient [13]. Erfanian et al. (2005)  have presented that simultaneous averaging method is one of the simplest ways to reduce the signal to noise ratio in quasi-periodic signals [17]. It is assumed that the EEG data signal has a specific nature, while we know that the noise is a statistical signal with normal distribution. In this method, the beginning of waveforms is specified and simultaneous averaging is applied. Next, the sum of all signals (for example, k signal) is calculated. The result of this process is a signal whose size is k time of the original waveform. Therefore, the estimated signal is achieved by scaling the obtained signal. The disadvantage of this approach is that by increasing the number of signals, the estimations of this method will not have appropriate efficiency [17]. Chaozhu et al. (2013) as well as Gholam-Hosseini et al. (1998) have used linear filtering and adaptive filtering techniques to reduce the effects of EOG and noise. Reducing the effect of ocular artifacts by adaptive filtering technique proves better results than linear filtering because data removal in the combined method is less than high-pass linear filtering. However, a part of signal is removed and it is not good to do so according to EEG signal sequence and brain signal interpreting [18, 19].

Reducing the effect of ocular artifacts (EOG) has been discussed By Ghanbari et al. in 2006. Vertical or horizontal eye movements are the main reasons to create potential differences. Vertical ocular signals occur in two states: vertical ocular signals (Veog) i.e. blinking and horizontal ocular signals (Heog) i.e. the eye movement on both sides. Blinking happens at low frequency and relatively high domain which is detectable due to large domain compared to EEG. The high-pass filter proposed by researchers is a kind of linear filtering. In this method, the components with cutoff frequency (Fc) are allowed to pass. In fact, the filter eliminates DC components and components with frequency lower than cutoff frequency. By increasing the order of filter, instability can be seen in filter response output that is not acceptable in EEG signal processing [20].

An adaptive filtering has been proposed by Shooshtari et al. in 2006. In this method, a slight delay has been applied in input that is multiplied by specific coefficients. The error between estimated artifact and reference artifact is calculated per multiplying signal at any moment. When this error reaches an acceptable value, the algorithm is stopped and the estimated artifact is subtracted from the raw signal. Like other algorithms, the disadvantage of this method is the process of synchronizing to receive EEG data and inappropriate rate of convergence. The process of algorithm will be in trouble by additional delay [21]. Lee et al. (2013) used wavelet analysis in a research. Fourier analysis is to decompose a signal into sine waves with different frequencies. In a similar manner, wavelet analysis is to decompose a signal into shifted and scaled versions of the original or main wavelet. Continuous Wavelet Transform (CWT) and Discrete Wavelet Transform (DWT) are two important processes in wavelet analysis [22]. Wavelet functions are the functions in which, most of their energy is concentrated at a small interval; they are quickly damped. Thus, with an appropriate selection of main wavelet, the compression can be done better. The method presented in this study is the wavelet transform through decomposition of waves into time. Regard high-speed of EOG signal separation from EEG, the estimation of signal separation is not accompanied by sufficient accuracy [23].

Kumar et al. (2008) used a method based on wavelet transform to remove EOG artifacts from EEG signals. Due to the nature of varying time, the signals associated with human body can be very efficient. Wavelet analysis is aimed at separating and isolating the structures with different time scales. Choosing appropriate wavelet and number of decomposition levels are selected based on the dominant frequency components in signal. Those parts of signals that are correlated with the frequency needed to classify the signals are kept in wavelet coefficients. Therefore, using this method depends on appropriate selection of wavelet. This is different for various data which cannot fully make the desired integrity and desirability [24].

Neural networks and fuzzy inference systems are proper nonlinear estimators that can be used. One of the efficient methods to be applied for this purpose is the combination of neural network and fuzzy system called fuzzy-neural network. Due to the above fact, an adaptive network based on RBFN and an adaptive FLN-RBFN network have been used to remove the artifacts from adaptive fuzzy inference network. Finally, it was found that FLN-RBFN network has a better performance than ANFIS and RBFN to remove artifacts. The method used to remove the artifacts must be such that it does not remove the original data of signals as much as possible and also remove the artifacts properly. With regard to comparison done on MSE of the method, FLN-RBFN method had the minimum MSE and the best performance. The advantage of this method is that there is need to set some parameters such as threshold level in wavelet transform which is heavily effective in noise removal performance. It detects the effect of artifacts in an adaptive manner and removes it. The disadvantage of FLN-RBFN method is that in addition to original signal, it needs to record the artifact signal with original signal.

In this research, the results of methods were compared and investigated separately and the result of eliminating artifact was observed in each method. 

Comparing the results of methods based on adaptive filter included an adaptive RBFN network, ANFIS and FLN-RBFN neuro-fuzzy interpretation network. It was indicated by MSE criteria that the function of FLN-RBFN neuro-fuzzy network with fewer number of neurons is better than adaptive ANFIS and RBFN networks.

Finally, in order to convert the written code to VHDL [25], the program was written online and taken to Simulink.
